# Solketal Removal from Aqueous Solutions Using Activated Carbon and a Metal–Organic Framework as Adsorbents

**DOI:** 10.3390/ma14226852

**Published:** 2021-11-13

**Authors:** Leticia Santamaría, Sophia A. Korili, Antonio Gil

**Affiliations:** INAMAT^2^, Departamento de Ciencias, Universidad Pública de Navarra, Campus de Arrosadía, 31006 Pamplona, Spain; leticia.santamaria@unavarra.es (L.S.); sofia.korili@unavarra.es (S.A.K.)

**Keywords:** solketal, emerging water pollutants, pharmaceutical compounds

## Abstract

The worldwide rise in biodiesel production has generated an excess of glycerol, a byproduct of the process. One of the most interesting alternative uses of glycerol is the production of solketal, a bioadditive that can improve the properties of both diesel and gasoline fuels. Even with its promising future, not much research has been performed on its toxicity in aqueous environments. In this work, solketal adsorption has been tested with two different commercial adsorbents: an activated carbon (Hydrodarco 3000) and a metal–organic framework (MIL-53). Diclofenac and caffeine were also chosen as emerging contaminants for comparison purposes. The effect of various parameters, such as the adsorbent mass or initial concentration of pollutants, has been studied. Adsorption kinetics with a better fit to a pseudo-second-order model, intraparticle diffusion, and effective diffusion coefficient were studied as well. Various isotherm equation models were employed to study the equilibrium process. The results obtained indicate that activated carbon is more effective in removing solketal from aqueous solutions than the metal–organic framework.

## 1. Introduction

The search for an alternative to petroleum-based fuels started decades ago due to their limited availability, high impact on the environment, and market fluctuations. Biodiesel has emerged as a great alternative to fossil fuels as it reduces chemical emissions such as unburned hydrocarbons (68%), polycyclic aromatic hydrocarbons (≈85%), and sulfur dioxide (100%) [1]. It can be produced from many renewable sources such as plant oils, animal fat, waste cooking oil, or photosynthetic algae [2]. In addition to its greener nature, its physical properties, close to those of fossil diesel, make it a great substitute, as the diesel engine and storage infrastructure do not need any modification [3]. Biodiesel is produced from triglycerides in a transesterification process, resulting in 90 wt.% methyl ester (biodiesel) and 10 wt.% glycerol as a byproduct. Despite the promising characteristics of biodiesel, some challenges need to be overcome, including the higher production costs when compared with fossil fuels, its refinement, and the surplus of glycerol, which has made the glycerol market price unstable [4]. Glycerol has traditionally been employed as an additive in food, pharmaceuticals, and tobacco [5]. However, with the development of biodiesel production, finding alternative uses for glycerol is vital to ensure that the global production of biodiesel is sustainable [2]. Glycerol can be modified through different reactions such as esterification, acetalization, and etherification [5,6,7,8,9]. Among these derivatives, ketals and acetals can be used as additives for gasoline and diesel fuel [10,11].

Solketal (2,2-dimethyl-1,3-dioxolane-4-methanol) has been revealed in recent years as one of the most interesting bioadditives due to its unique properties. Solketal improves the cold flow properties of diesel fuel and the oxidation stability, gum formation, and octane number of gasoline fuel [12]. However, there is limited research performed regarding its toxicity: an aquatox fish test showed that solketal (LC_50_ = 3.162 ppm) had less environmental toxicity than methyl tertiary-butyl ether, MTBE, a common fuel additive (LC_50_ < 1000) [13,14]. These studies used solketal as a solvent for zebrafish bioassays, finding that embryos and larvae had problems tolerating it even at the lowest concentration (0.5%). Concentrations of 1% and above induced developmental delay, impaired circulation and motility in embryos, and caused a loss of posture and brain necrosis in larvae.

Considering that solketal has been proven to cause harm in a water environment, a study of possible adsorbents in aqueous media needs to be carried out in case of the accidental release of solketal into the environment due to an oil spill. The present study tests the effectiveness of two commercial adsorbents for the removal of solketal: a granular activated carbon (AC) and a metal–organic framework (MOF). Two emerging contaminants (diclofenac sodium and caffeine) were also chosen for comparison purposes [15,16,17,18,19]. The efficacy of several MOFs for the adsorption of pharmaceutical compounds has been tested before [20,21,22] as well as that of AC [23,24,25,26]; however, to our best knowledge, their solketal sorption capacity is yet to be tested. In addition, various kinetic and equilibrium models were assessed for the adsorption of these three organic molecules on the two adsorbents.

## 2. Experimental Procedure

### 2.1. Materials and Characterization Techniques

The AC used in this work, Hydrodarco 3000, was kindly supplied by Cabot Corporation (Boston, MA, USA). This granular, acid-washed carbon is produced by the steam activation of coal and has excellent adsorption capacity for organic molecules. The MOF, called Basolite A100, is a Sigma-Aldrich trademark for the MIL-53(Al) MOF. This adsorbent has a structure built up from infinite chains of corner-sharing AlO_4_(OH)_2_ octahedra connected through 1,4-benzenedicarboxylate ligands.

The chemical reagents used as adsorbates (caffeine, diclofenac sodium, and solketal) were purchased from Sigma-Aldrich (Madrid, Spain) with purity above 97%; their chemical structures/physicochemical characteristics are displayed in Table 1.

N_2_ adsorption was performed at 77 K with a Micromeritics ASAP 2010 adsorption analyzer (Micromeritics, Atlanta, GA, USA). The samples (0.4 g) were degassed under vacuum before measurement at 423 K for 24 h. The specific surface area (*S_BET_*) was evaluated by the Brunauer Emmett Teller (BET) method in the range between 0.05 and 0.20 relative pressure. The external surface area (*S_ext_*) and the micropore volumes (*V_μp_*) were also estimated using the t-plot method.

### 2.2. Adsorption Procedure

The adsorption experiments of solketal, diclofenac, and caffeine were carried out under batch-adsorption conditions. Kinetic tests were performed to study both the effect of drug concentration and the adsorbent dose. In the case of diclofenac and caffeine, the effect of the drug concentration was studied by adding 20 or 75 mg of the adsorbent to 200 cm^3^ of solutions containing several drug concentrations (10, 15, and 20 mg/dm^3^). In the case of solketal, due to its low signal on the UV spectrophotometer, larger concentrations were chosen: 1.5 g of the adsorbent to a 50, 75, or 100 g/dm^3^ solketal concentration. The adsorbent dose was examined by using various amounts of each adsorbent: 10, 20, and 30 mg for diclofenac and caffeine with AC; 50, 75, and 100 mg for diclofenac and caffeine with MOF; or 1, 1.5, and 2 g for solketal in a 200 cm^3^ solution with a drug concentration of 15 mg/dm^3^ or 75 g/dm^3^, respectively. Experiments were performed with no pH modification at 298 K, with samples being shaken on a stirring plate throughout the duration of the experiments. Samples of the solution were taken at various time intervals until equilibrium was attained, up to 400 min. The contaminant concentration in the filtered solutions (0.45 μmol, Durapore) was determined by a Jasco V-730 UV–Vis spectrophotometer (JASCO Analitica, Madrid, Spain). Maximum adsorption wavelengths used were 276.5 nm for diclofenac, 273.5 nm for caffeine, and 239 nm for solketal. The amount of pollutant adsorbed by AC and MOF was estimated by subtracting the initial and remaining concentrations, using the following equation:

(1) $$q_{t,e}=\frac{V\cdot \left(C_{0}-C_{t,e}\right)}{m}$$

where *C*_0_ (mg/dm^3^) is the initial concentration and *C_t_* is the concentration at a time *t* of the pollutant in solution, *m* (g) is the adsorbent mass, and *V* (cm^3^) is the volume of the solution.

In order to study the adsorption capacity of both the MOF and AC on the three pollutants, equilibrium studies were also performed. Drug solutions of various concentrations (between 10 and 500 mg/dm^3^ in the case of the drugs and between 10 and 500 g/dm^3^ for solketal) were placed in 10 cm^3^ glass tubes with 10 mg of either of the adsorbents (AC or MOF) and shaken for 7 h. Drug concentrations of the filtered samples were calculated following UV-visible spectrophotometry using Equation (1) (*C_e_*, in either mg/dm^3^ or g/dm^3^, was the drug concentration at equilibrium). All adsorption experiments were performed twice, and the reported results are the average of these two measurements.

## 3. Results and Discussion

### 3.1. Characterization of the Adsorbents

The nitrogen adsorption–desorption data for samples at 77 K showed that the adsorption isotherm for AC was of type II in the Brunauer, Deming, Deming, and Teller (BDDT) classification [27] with a hysteresis loop of type IIIb in the Rouquerol et al. classification [28], indicating that the adsorbent is a mesoporous material. The BET-specific surface area of the AC was 578 m^2^/g, with a 0.564 cm^3^/g total pore volume obtained at a relative pressure of 0.98. The t-plot method was used to estimate the external specific surface area and micropore volume, which were 159 m^2^/g and 0.206 cm^3^/g, respectively. The MOF adsorption isotherm was also found to be type II in the BDDT classification with a 465 m^2^/g BET specific surface, an external specific surface area of 143 m^2^/g, a total 1.05 cm^3^/g pore volume, and 0.13 cm^3^/g micropore volume.

### 3.2. Adsorption Experiments

**Figure 1:**
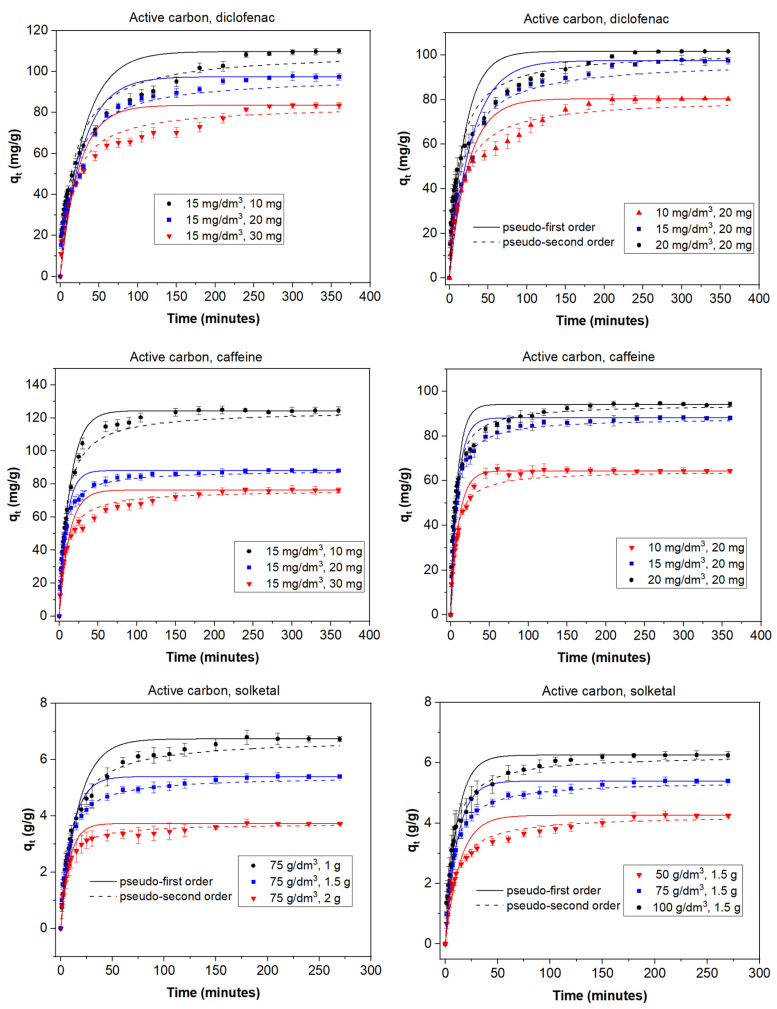
Kinetic adsorption data for diclofenac, caffeine, and solketal on AC using various absorbate concentrations (10–20 mg/dm^3^; 50–100 g/dm^3^) and adsorbent amounts (10–30 mg; 1–2 g). T = 298 K.

**Figure 2:**
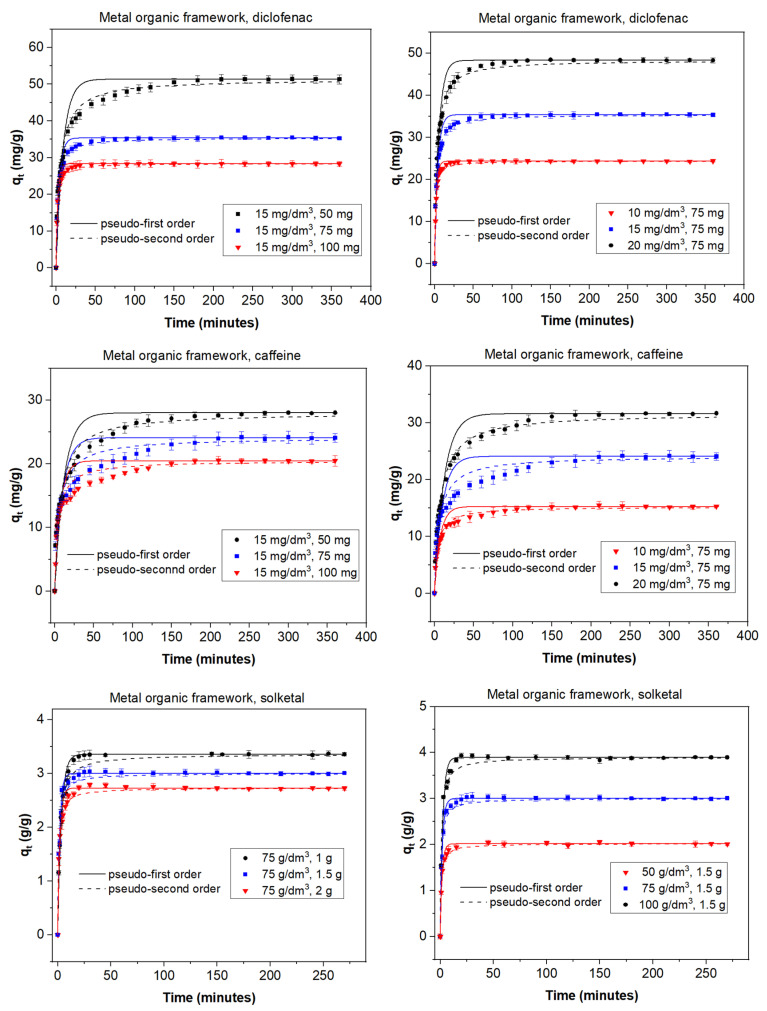
Kinetic adsorption data for diclofenac, caffeine, and solketal on the MOF using various adsorbate concentrations (10–20 mg/dm^3^; 50–100 g/dm^3^) and adsorbent amounts (10–30 mg; 1–2 g). T = 298 K.

The adsorption process can be affected by several factors that play an important role; among them, the adsorbent dose and the initial concentration of adsorbate are of great influence on the final result. In order to study the role of the adsorbent dose, various doses were used depending on both the adsorbent and the adsorbate. With diclofenac and caffeine as adsorbates (15 mg/dm^3^) and activated carbon as the adsorbent, doses between 10 and 30 mg were studied. When solketal was the adsorbate, AC doses ranged from 1 to 2 g. The results are summarized in the first column of Figure 1. It can be seen that the adsorption capacity of AC was similar for diclofenac and caffeine and much greater in the case of solketal, where the AC adsorption capacity is expressed in g/g adsorbent. In MOF experiments, doses of 50, 75, and 100 mg of the adsorbent on a 15 mg/dm^3^ concentration of diclofenac or caffeine were analyzed; the same doses of solketal that were used with the AC were replicated for the MOF experiments. Results are shown in the first column of Figure 2. When MOF is used, its diclofenac adsorption capacity is greater than that of caffeine. As a whole, the adsorption capacity of MOF is, in all cases, lower than when AC is used as an adsorbent. In all experiments, the adsorption capacity in equilibrium increased with an increasing amount of adsorbent dose. For example, in the case of AC and diclofenac (15 mg/dm^3^), the amount adsorbed increased between 35.9% and 83.8% when the amount of adsorbent increased from 10 to 30 mg. This result is likely due to particle interactions and aggregation of the adsorption sites, which causes a decrease in the effective adsorbent surface area available to the organic contaminants.

To study the effect of the initial adsorbate concentration, various concentrations of the organic molecules were chosen: between 10 and 20 mg/dm^3^ for diclofenac and caffeine or between 50 and 100 g/dm^3^ for solketal. The experiments were performed with a fixed amount of adsorbent at 298 K with no pH modification, and equilibrium was achieved in less than 400 min. Results are shown in the second columns of Figure 1 for AC and Figure 2 for MOF. In all experiments, a higher initial adsorbate concentration results in a higher adsorption capacity. For example, in the case of MOF (75 mg) and caffeine, the amount adsorbed increased by between 56.0% and 59.3% when the concentration of adsorbate increased from 10 to 20 mg/dm^3^. This can be due to the fact that each adsorbent has the capacity to adsorb a fixed quantity of the pollutant; thus, an increase in the adsorbate concentration produces an increase in the adsorption capacity until this capacity is reached. Additionally, the concentration gradient, acting as a force that overcomes mass-transfer resistance of the adsorbate between the aqueous and solid phases, could be partially responsible. A higher initial concentration leads to a higher adsorption capacity as it provides a greater driving force.

### 3.3. Kinetic Models

The kinetic modeling was studied using three different rate equations: pseudo-first (2), pseudo-second-order (3), and intraparticle-diffusion models (6). These types of kinetic modeling approaches are considered to indicate a simple relationship between the adsorption performance and operating conditions and show how the average adsorbent phase concentration (*q*) changes with adsorption time. The first-order rate equation can describe the initial phase of the adsorption process, although, as adsorption proceeds, the adsorption data may deviate from the fitted curve. The second-order rate equation suggests that chemisorption is the speed control mechanism. These equations have been used frequently to analyze adsorption data obtained from various experiments with various types of adsorbates and adsorbents.

(2) $$\frac{dq}{dt}=k_{1}\left(q_{e}-q_{t}\right)$$

(3) $$\frac{dq}{dt}=k_{2}{\left(q_{e}-q_{t}\right)}^{2}$$

where *q_e_* is the amount of adsorbed solute at equilibrium and *q_t_* at a time *t* (mg/g for diclofenac and caffeine and g/g for solketal), and *k*_1_ and *k*_2_ are the reaction rate constants of pseudo-first and pseudo-second-order models, respectively.

Integrating the equations between *t =* 0 and time *t*, the following equations are obtained:

(4) $$q_{t}=q_{e}\cdot (1-\exp(-k_{1}\cdot t)$$

(5) $$q_{t}=\frac{k_{2}\cdot q_{e}^{2}\cdot t}{1+k_{2}\cdot q_{e}\cdot t}$$

The experimental data were tested using the OriginPro program (2018 version) in order to analyze the transport of organic molecules onto the adsorbent particles. The parameters of the equations were estimated by fitting the models to the experimental data by non-linear regression analysis.

The Weber–Morris linear representations for describing the kinetics of sorption at solid/solution interfaces controlled by intraparticle diffusion was also used [29]:

(6) $$q_{t}=k_{3}\cdot t^{0.5}$$

where *k*_3_ (mg/g·min^0.5^ or g/g·min^0.5^) is the intraparticle-diffusion rate constant. Assuming constant diffusion through adsorbent pores [30], if the Weber–Morris plot of *q_t_* versus *t*^0.5^ gives a straight line, this means that the sorption process is only controlled by intraparticle diffusion. In the same way, two or more steps influence the sorption process if the data exhibit multi-linear plots [31,32].

Intraparticle diffusion was also studied with a fractional approach to the equilibrium and used to estimate the effective diffusion coefficient [15,33]:

(7) $$F\left(t\right)=\frac{C_{0}-C_{t}}{C_{0}-C_{e}}=\sqrt{1-exp\left(-\frac{\pi^{2}\cdot D\cdot t}{r^{2}}\right)}$$

where *D* (m^2^/s) is the intraparticle-diffusion coefficient and *r* (m) is the particle size radius, assuming a perfect sphere.

The kinetic behavior of the adsorption processes is described in Figure 1 and Figure 2 as well as Tables S1 and S2. The pseudo-second-order linear reaction better describes the process in all cases. When AC is used as the adsorbent (Figure 1, Table S1 (Supplementary Information)), the adsorption rate is faster for solketal, as it has the highest values. The adsorption rate constant is similar for caffeine and diclofenac, although caffeine figures are slightly greater. An analysis of the change as the adsorbate increases in concentration from 10 mg/dm^3^ to 20 mg/dm^3^ shows no significant change in the *k*_2_ values but displays a slight decrease. However, an increase in adsorbent mass from 10 to 30 mg reveals an increase in the adsorption rate constant. When the MOF was used as the adsorbent (Figure 2, Table S2), the adsorption rate was, in general, faster than for AC, as *k*_2_ values were greater and equilibrium was achieved faster. A comparison between adsorbates shows, again, a much greater adsorption rate for solketal than the pharmaceutical compounds, where diclofenac exhibits a slightly larger *k*_2_ value compared to caffeine. An increase in the concentration of adsorbate shows a decrease in the adsorption rate constant and, as is also the case in the AC results, a decrease in the *k*_2_ values.

**Figure 3:**
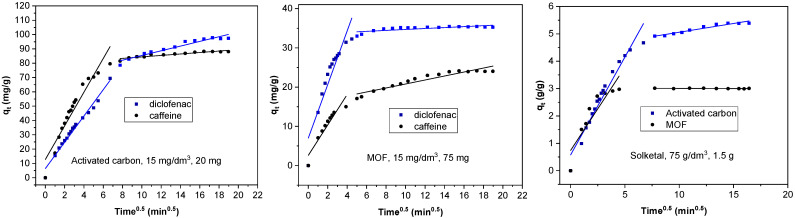
Intraparticle-diffusion model for the adsorption of organic molecules on the AC and MOF.

As illustrated in Figure 1 and Figure 2, equilibrium was achieved faster when the MOF was used as the adsorbent, although the total amount adsorbed was always less. This is more obvious in the case of diclofenac and, particularly, solketal, where the MOF displayed a very rapid initial adsorption in the first 20 min. The adsorption process is thus separated into two stages: an initial stage that is short and displays a faster adsorption rate as there are a larger number of free and stronger active sites, followed by a slower and longer uptake period in which repulsive forces appear between the already adsorbed organic molecules and the ones in the bulk phase, preventing them from being adsorbed. This is further studied with the intraparticle diffusion of the organic molecules, presented in Figure 3 and Table S3. The first slope of the process, *k*_3_′, represents the external mass transfer and the fast distribution of the organic molecules on the most superficial layer of the adsorbent. The second slope, *k*_3_*″*, is related to the intraparticle diffusion into the more internal active sites of the adsorbent. As can be seen in Table S3, the second slope in the case of the MOF is very small (namely in the case of diclofenac and solketal), as almost all the adsorbate uptake takes place in the first adsorption stage. However, the AC adsorption processes have a non-negligible second slope. This could be related to a major number of internal active sites as the AC has double the microscope volume of the MOF. The adsorption process in porous adsorbents was also examined with the estimation of the effective diffusion coefficient by applying a fractional approach to the equilibrium, with results shown in Table S4. The lower value of *D/r*^2^, intraparticle diffusion, when AC was the adsorbent, can indicate a slower uptake by the internal active sites, which is related to a greater *k*_3_*″* value. The MOF *D/r*^2^ values are larger, which facilitates the internal sites’ adsorption process. The general trend shows that as the adsorbent amount increases, so does the effective diffusion coefficient.

### 3.4. Adsorption Isotherms

Various models can be considered to study the experimental equilibrium data and better understand the behavior between pollutants and adsorbents. Langmuir (Equation (8)) and Freundlich (Equation (9)) were chosen as two-parameter isotherms, and Toth (Equation (10)) as a three-parameter isotherm. Being first designed to describe gas-solid phase adsorption, the Langmuir isotherm balances the relative rate of adsorption and desorption. It assumes monolayer adsorption onto a surface that contains a finite number of sites with uniform characteristics [34]. The Freundlich isotherm was designated to be applicable to an adsorption process that takes place on heterogeneous surfaces. The expression that the isotherm gives describes the surface heterogeneity as well as the distribution and energies of the active sites [35]. The Toth isotherm is another empirical modification of the Langmuir equation, with the purpose of reducing the difference between experimental data and the predicted value of equilibrium data. This model is most valuable for the description of heterogeneous adsorption systems which satisfy both the low- and high-end boundary of adsorbate concentration. The third parameter added, *m_T_*, characterizes the heterogeneity of the adsorption system; when *m_T_* = 1, this equation reduces to the Langmuir isotherm equation. If this parameter deviates further away from unity (1), then the system is said to be heterogeneous [36].

(8) $$q_{e}=\frac{k_{L}\cdot q_{L}\cdot C_{e}}{1+k_{L}\cdot C_{e}}$$

(9) $$q_{e}=k_{F}\cdot C_{e}^{1/m_{F}}$$

(10) $$q_{e}=\frac{k_{T}\cdot q_{T}\cdot C_{e}}{{\left[1+{\left(k_{T}\cdot C_{e}\right)}^{m_{T}}\right]}^{1/m_{T}}}$$

where *q_e_* (mg or g of adsorbate/g of adsorbent) is the amount adsorbed, *C_e_* (mg or g/g) is the monolayer capacity, *k_L_* is related to the adsorption capacity, *k_F_* is the equilibrium constant, 1/*m_F_* is the adsorption intensity (also indicates the heterogeneity of the adsorbate sites), and *k_T_* is the Toth isotherm constant.

The three isotherm equations were adjusted to the experimental data for solketal, diclofenac, and caffeine, with AC and the MOF as adsorbents. The results are represented in Figure S1, and the calculated constants for the equations are shown in Table 2 (AC) and Table 3 (MOF). As defined by the Giles classification, the isotherms have an L-class shape, typical of systems with high adsorbate–adsorbent attraction [37]. Of the three models tested, the Toth equation gives the best adjustment to the results, which makes sense as it is an empirical modification of the Langmuir equation to better fit the results. It is difficult to compare these results with other published work, as often only the Freundlich and Langmuir equations are tested, with the latter generally giving the best results for the analysis of pharmaceuticals, pesticides, herbicides, and organic compounds [25].

### 3.5. Mechanism of Adsorption on the Adsorbents

Various types of interactions have been reported between the surface of the adsorbents and organic contaminants to explain the removal capacity of the materials: hydrophobic interactions, π–π stacking interactions, van der Waals forces, electrostatic interactions, and hydrogen bonding interactions that can occur either individually or simultaneously [38,39].

The results of adsorption suggest that the two commercial adsorbents (AC and MOF) show an affinity for the three organic compounds studied. The attractive or repulsive interactions between the adsorbent surface and the molecule are affected by the surface chemical characteristics of the materials. The net charge of the adsorbent surface in solution is characterized by the point of zero charge. In the case of the activated carbon, a value of 4.5 was found in previous work [26], indicating that the material has a negative charge under the adsorption conditions of this study. Therefore, an electrostatic attraction between the negatively charged surface and the organic molecules could be possible. As such, the effect of van der Waals and π–π interactions in the removal of the organic molecules cannot be neglected under the experimental conditions used. The MOF (MIL-53(Al)) has a structure built up from infinite chains of corner-sharing AlO_4_(OH)_2_ octahedra connected through 1,4-benzenedicarboxylate ligands. This structure may present similarities with the surface properties of AC; in this case, it can also be proposed that the same type of interactions described above will occur.

## 4. Conclusions

In this study, solketal adsorption, together with diclofenac and caffeine for comparison, has been tested due to a lack of data regarding its removal from water by adsorption. AC and a MOF have been tested as adsorbents; AC gave the best results (71% of solketal adsorbed), although the MOF was faster in achieving equilibrium (25 min). The effect of various parameters, such as the adsorbent mass or initial concentration of pollutant, was studied. Adsorption kinetics analyses revealed a better fit to a pseudo-second-order model, where the adsorption process was separated into two stages. The AC gave a lower value in the intraparticle diffusion, which may indicate a slower uptake by the internal active sites. Adsorption isotherms were also fitted to the experimental data, and the three-parameter Toth equation gave a better agreement between the experimental and theoretical data than the Langmuir and Freundlich equations. Overall, both adsorbents have been proven to be very effective for solketal adsorption, being able to remove up to 71% of the solketal present from aqueous solutions.

## Figures and Tables

**Table 1:** Chemical structures/physicochemical characteristics of the adsorbates.

Name	Structure and Characteristics	
Caffeine	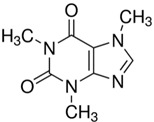	C_8_H_10_N_4_O_2_
		m = 194.19 g/mol
		pk_a_ = 10.4
Diclofenac sodium	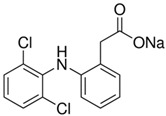	C_14_H_10_Cl_2_NNaO_2_
		m = 296.15 g/mol
		pk_a_ = 4.15
Solketal	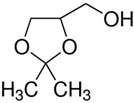	C_6_H_12_O_3_
		m = 132.16 g/mol
		pk_a_ = 14.20

**Table 2:** Freundlich, Langmuir, and Toth parameters for adsorption by the AC. Equilibrium time = 7 h, T = 298 K.

	Solketal *	Diclofenac	Caffeine
Freundlich			
*k_F_* (dm^3^/mg)*^mF^*	3.7	4.6	12.6
*m_F_*	1.82	2.05	2.63
χ^2^	726	449	3581
R	0.93	0.95	0.76
Langmuir			
*q_L_* (mg/g)	83.7	68.4	82.8
*k_L_* (dm^3^/mg)	0.013	0.021	0.058
χ^2^	331	112	2128
R	0.97	0.99	0.86
Toth			
*q_T_* (mg/g)	90.3	2122	79.6
*k_T_* (dm^3^/mg)	0.013	0.27	0.37
*m_T_*	0.66	0.13	0.61
χ^2^	121	244	496
R	0.99	0.998	0.991

* *q* (g_adsorbate_/g_adsorbent_); *k* (dm^3^/g_adsorbate_).

**Table 3:** Freundlich, Langmuir, and Toth parameters for adsorption by the MOF. Equilibrium time = 7 h, T= 298 K.

	Solketal *	Diclofenac	Caffeine
Freundlich			
*k_F_* (dm^3^/mg)*^mF^*	2.57	6.13	7.87
*m_F_*	1.76	2.60	2.41
χ^2^	377	410	1428
R	0.91	0.91	0.78
Langmuir			
*q_L_* (mg/g)	56.9	43.0	55.5
*k_L_* (dm^3^/mg)	0.016	0.050	0.063
χ^2^	189	109	833
R	0.96	0.98	0.88
Toth			
*q_T_* (mg/g)	124.4	78.91	71.4
*k_T_* (dm^3^/mg)	0.017	0.26	0.51
*m_T_*	0.46	0.35	0.42
χ^2^	219	170	401
R	0.99	0.998	0.99

* *q* (g_adsorbate_/g_adsorbent_); *k* (dm^3^/g_adsorbate_).

## Data Availability

Not applicable.

## Supplementary Materials

The following are available online at www.mdpi.com/article/10.3390/ma14226852/s1, Figure S1: Experimental results (scatter) and isotherm adjustment to Langmuir, Freundlich and Toth models for diclofenac, caffeine and solketal adsorption on the AC (first column) and MOF (second column), Table S1: Pseudo-first and pseudo-second-order parameters for the adsorption of organic molecules by the activated carbon., Table S2: Pseudo-first and pseudo-second-order parameters for the adsorption of organic molecules by the metal organic framework, Table S3: Intraparticle rate parameters for the adsorption of organic molecules by the AC and MOF, Table S4: Effective diffusion coefficients for the adsorption of organic molecules by the AC and MOF.
