# Supplementary material for: Solketal Removal from Aqueous Solutions Using Activated Carbon and a Metal–Organic Framework as Adsorbents
Source: Materials (Basel). 2021 Nov 13;14(22):6852. doi: 10.3390/ma14226852 (PMC8623111; doi:10.3390/ma14226852)
Supplement: Supplementary file 1 [file materials-14-06852-s001.zip › materials-1451248-SI.pdf]

materials

MDPI

Supplementary Information

Solketal Removal from Aqueous Solutions Using Activated Carbon and a Metal–Organic Framework as Adsorbents

Leticia Santamaría, Sophia A. Korili and Antonio Gil *

INAMAT[2], Departamento de Ciencias, Universidad Pública de Navarra, Campus de Arrosadía, 31006 Pamplona, Spain; leticia.santamaria@unavarra.es (L.S.); sofia.korili@unavarra.es (S.A.K.)

* Correspondence: andoni@unavarra.es; Tel.: +34-948-169-602

Table S1. Pseudo-first and pseudo-second-order parameters for the adsorption of organic molecules by the activated carbon.

Sample		Pseudo-First Order						Pseudo-Second Order				
		**10 mg/dm³ 20 mg**	**15 mg/dm³ 20 mg**	**20 mg/dm³ 20 mg**	**15 mg/dm³ 10 mg**	**15 mg/dm³ 30 mg**		**10 mg/dm³ 20 mg**	**15 mg/dm³ 20 mg**	**20 mg/dm³ 20 mg**	**15 mg/dm³ 10 mg**	**15 mg/dm³ 30 mg**
**Diclofenac**	k_1 (1/min)	0.041	0.036	0.051	0.034	0.042	k_2 (1/min)	8.73×10^{-4}	6.48×10^{-4}	8.45×10^{-4}	5.43×10^{-4}	8.29×10^{-4}
	χ^2	1836	2044	3388	1372	2436	χ^2	439	823	815	1128	520
	R	0.95	0.96	0.93	0.93	0.93	R	0.99	0.99	0.98	0.98	0.99
**Caffeine**	k_1 (1/min)	0.098	0.108	0.117	0.072	0.079	k_2 (1/min)	2.68×10^{-3}	2.10×10^{-3}	2.12×10^{-3}	9.61E-4	1.69×10^{-3}
	χ^2	319	943	1646	1096	1470	χ^2	216	101	199	533	240
	R	0.98	0.97	0.95	0.99	0.94	R	0.99	0.997	0.995	0.994	0.991
		**50 g/dm³ 1.5 g**	**75 g/dm³ 1.5 g**	**100 g/dm³ 1.5 g**	**75 g/dm³ 1 g**	**75 g/dm³ 2 g**		**50 g/dm³ 1.5 g**	**75 g/dm³ 1.5 g**	**100 g/dm³ 1.5 g**	**75 g/dm³ 1 g**	**75 g/dm³ 2 g**
**Solketal**	k_1 (1/min)	0.072	0.089	0.092	0.060	0.12	k_2 (1/min)	0.027	0.027	0.024	0.014	0.054
	χ^2	4	3.7	5.8	6	1.75	χ^2	0.52	0.40	0.87	0.97	0.18
	R	0.95	0.97	0.97	0.97	0.96	R	0.993	0.997	0.995	0.996	0.996

Table S2. Pseudo-first and pseudo-second-order parameters for the adsorption of organic molecules by the metal organic framework.

Sample		Pseudo-First Order						Pseudo-Second Order				
		**10 mg/dm³ 75 mg**	**15 mg/dm³ 75 mg**	**20 mg/dm³ 75 mg**	**15 mg/dm³ 50 mg**	**15 mg/dm³ 100 mg**		**10 mg/dm³ 75 mg**	**15 mg/dm³ 75 mg**	**20 mg/dm³ 75 mg**	**15 mg/dm³ 50 mg**	**15 mg/dm³ 100 mg**
**Diclofenac**	k_1 (1/min)	0.044	0.245	0.178	0.011	0.041	k_2 (1/min)	0.040	0.014	6.94×10^{-3}	3.80×10^{-3}	0.032
	χ^2	14	130	263	593	40.4	χ^2	10.2	9.3	25	125	3.82
	R	0.991	0.97	0.97	0.94	0.98	R	0.993	0.996	0.997	0.99	0.998
**Caffeine**	k_1 (1/min)	0.14	0.100	0.081	0.082	0.14	k_2 (1/min)	0.016	6.92×10^{-3}	4.31×10^{-3}	4.96×10^{-3}	0.011
	χ^2	46	254	177	220	140	χ^2	7.7	76	27.9	54	34
	R	0.95	0.87	0.96	0.93	0.90	R	0.991	0.96	0.994	0.98	0.98
		**50 g/dm³ 1.5 g**	**75 g/dm³ 1.5 g**	**100 g/dm³ 1.5 g**	**75 g/dm³ 1 g**	**75 g/dm³ 2 g**		**50 g/dm³ 1.5 g**	**75 g/dm³ 1.5 g**	**100 g/dm³ 1.5 g**	**75 g/dm³ 1 g**	**75 g/dm³ 2 g**
**Solketal**	k_1 (1/min)	0.56	0.51	0.41	0.29	0.48	k_2 (1/min)	0.56	0.36	0.234	0.18	0.39
	χ^2	0.07	0.20	0.44	0.43	0.42	χ^2	0.032	0.27	0.30	0.29	0.09
	R	0.991	0.991	0.99	0.99	0.98	R	0.996	0.99	0.992	0.99	0.994

Table S3. Intraparticle rate parameters for the adsorption of organic molecules by the AC and MOF.

Sample		Active Carbon					MOF				
		**10 mg/dm^3 20 mg**	**15 mg/dm^3 20 mg**	**20 mg/dm^3 20 mg**	**15 mg/dm^3 10 mg**	**15 mg/dm^3 30 mg**	**10 mg/dm^3 75 mg**	**15 mg/dm^3 75 mg**	**20 mg/dm^3 75 mg**	**15 mg/dm^3 50 mg**	**15 mg/dm^3 100 mg**
**Diclofenac**	K_3'(mg/g·min$^{0.5}$)	8.54	9.18	10.31	10.27	8.71	5.03	7.00	9.14	8.32	5.64
	R	0.99	0.99	0.97	0.99	0.99	0.80	0.94	0.96	0.97	0.89
	K_3''(mg/g·min$^{0.5}$)	1.99	1.64	2.00	2.94	2.02	0.01	0.12	0.27	0.71	0.03
	R	0.92	0.97	0.95	0.98	0.98	0.55	0.77	0.77	0.91	0.74
**Caffeine**	K_3'(mg/g·min$^{0.5}$)	9.40	11.76	11.64	17.14	8.92	2.64	3.35	4.93	3.95	3.30
	R	0.99	0.96	0.94	0.98	0.95	0.97	0.93	0.99	0.97	0.91
	K_3''(mg/g·min$^{0.5}$)	0.12	0.51	0.77	0.77	1.13	0.19	0.51	0.52	0.52	0.35
	R	0.63	0.93	0.91	0.76	0.95	0.86	0.95	0.90	0.90	0.93
		**50 g/dm^3 1.5 g**	**75 g/dm^3 1.5 g**	**100 g/dm^3 1.5 g**	**75 g/dm^3 1 g**	**75 g/dm^3 2 g**	**50 g/dm^3 1.5 g**	**75 g/dm^3 1.5 g**	**100 g/dm^3 1.5 g**	**75 g/dm^3 1 g**	**75 g/dm^3 2 g**
**Solketal**	K_3'(mg/g·min$^{0.5}$)	0.41	0.71	0.80	0.84	0.50	0.49	0.72	0.86	0.83	0.64
	R	0.97	0.97	0.96	0.98	0.97	0.90	0.92	0.92	0.97	0.92
	K_3''(mg/g·min$^{0.5}$)	0.093	0.064	0.067	0.10	0.055	5E-4	0.002	0.001	0.002	0.002
	R	0.97	0.97	0.91	0.94	0.95	0.10	0.61	0.19	0.72	0.68

Table S4. Effective diffusion coefficients for the adsorption of organic molecules by the AC and MOF.

Sample		Active Carbon					MOF				
		**10 mg/dm³ 20 mg**	**15 mg/dm³ 20 mg**	**20 mg/dm³ 20 mg**	**15 mg/dm³ 10 mg**	**15 mg/dm³ 30 mg**	**10 mg/dm³ 75 mg**	**15 mg/dm³ 75 mg**	**20 mg/dm³ 75 mg**	**15 mg/dm³ 50 mg**	**15 mg/dm³ 100 mg**
**Diclofenac**	D/r^2 (1/s)	2.66 × 10^{-5}	2.41 × 10^{-5}	3.26 × 10^{-5}	2.21 × 10^{-5}	2.56 × 10^{-5}	4.03 × 10^{-4}	2.08 × 10^{-4}	1.45 × 10^{-4}	7.85 × 10^{-5}	3.78 × 10^{-4}
	χ^2	0.031	0.020	0.050	0.038	0.064	0.0069	0.017	0.017	0.055	0.0096
	R	0.994	0.997	0.99	0.993	0.99	0.998	0.994	0.995	0.99	0.996
**Caffeine**	D/r^2 (1/s)	7.61 × 10^{-5}	8.05 × 10^{-5}	8.55 × 10^{-5}	5.19 × 10^{-5}	5.21 × 10^{-5}	1.02 × 10^{-4}	5.97 × 10^{-5}	5.66 × 10^{-5}	5.38 × 10^{-5}	9.66 × 10^{-5}
	χ^2	0.008	0.015	0.042	0.014	0.066	0.052	0.17	0.024	0.058	0.15
	R	0.998	0.996	0.99	0.997	0.98	0.985	0.95	0.994	0.99	0.95
		**50 g/dm³ 1.5 g**	**75 g/dm³ 1.5 g**	**100 g/dm³ 1.5 g**	**75 g/dm³ 1 g**	**75 g/dm³ 2 g**	**50 g/dm³ 1.5 g**	**75 g/dm³ 1.5 g**	**100 g/dm³ 1.5 g**	**75 g/dm³ 1 g**	**75 g/dm³ 2 g**
**Solketal**	D/r^2 (1/s)	4.73 × 10^{-5}	6.35 × 10^{-5}	6.45 × 10^{-5}	4.15 × 10^{-5}	8.9 × 10^{-5}	4.95 × 10^{-4}	4.61 × 10^{-4}	3.72 × 10^{-4}	2.56 × 10^{-4}	3.88 × 10^{-4}
	χ^2	0.049	0.015	0.028	0.013	0.045	0.0057	0.014	0.012	0.0092	0.012
	R	0.99	0.996	0.993	0.998	0.99	0.997	0.993	0.995	0.996	0.996

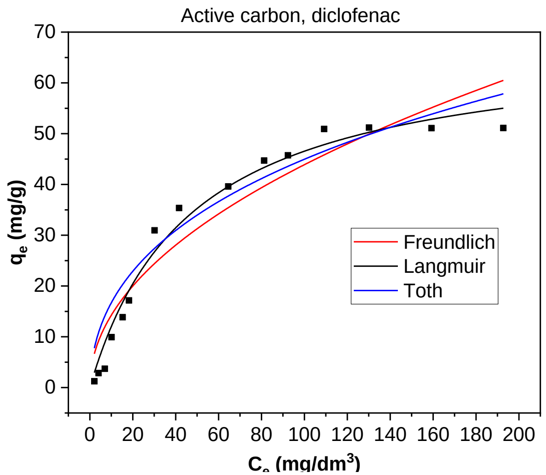

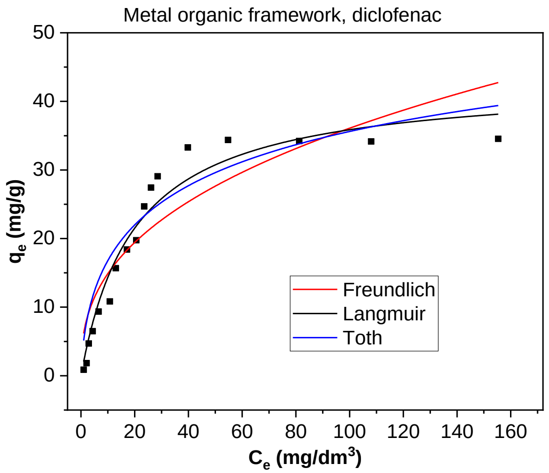

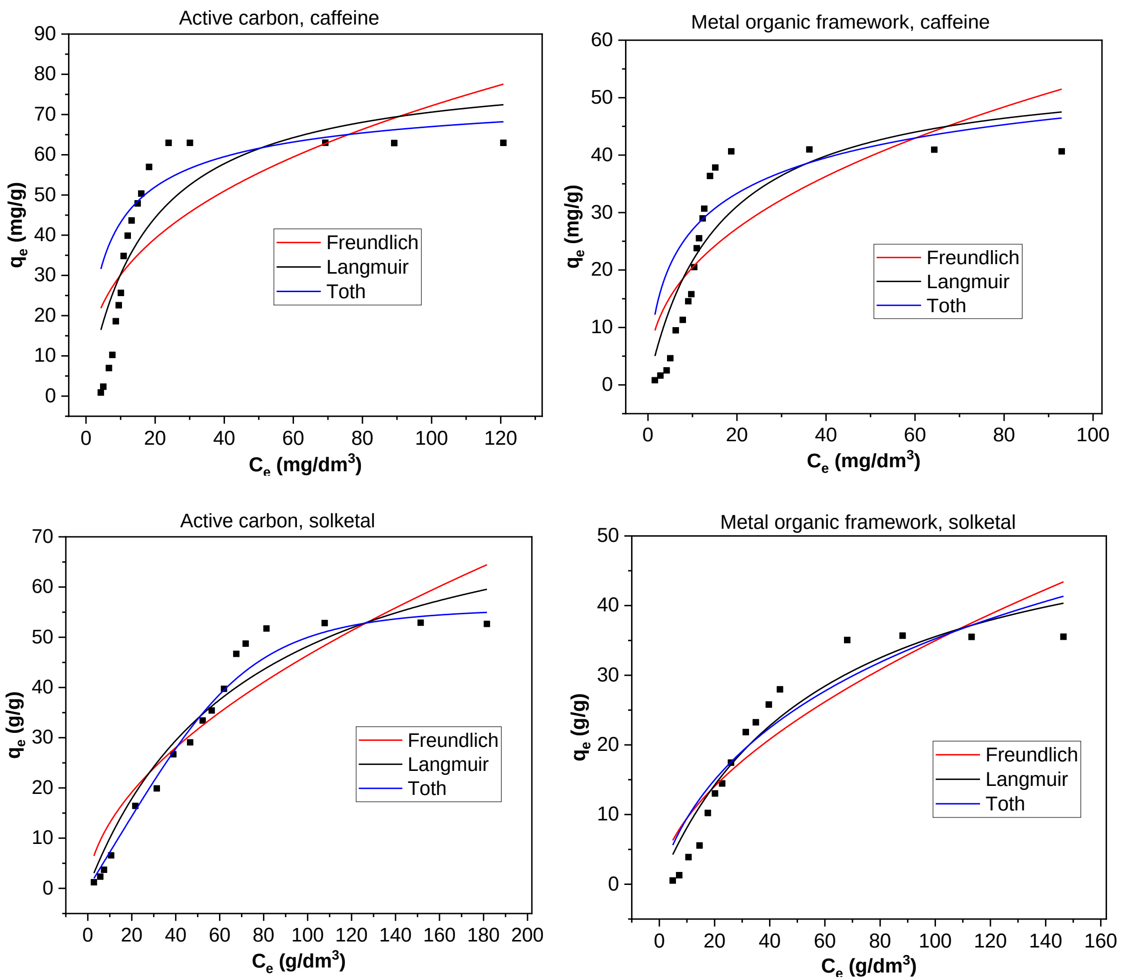

Figure S1. Experimental results (scatter) and isotherm adjustment to Langmuir, Freundlich and Toth models for diclofenac, caffeine and solketal adsorption on the AC (first column) and MOF (second column).
